# Effects of Pictogram and Typeface Complexity on Visual Attention: Eye-Tracking Study

**DOI:** 10.3390/jemr19040073

**Published:** 2026-07-07

**Authors:** Klementina Možina, Dorotea Kovačević, Petra Buljat, Iva Šarčević, Barbara Blaznik, Tanja Medved, Maja Brozović

**Affiliations:** 1 Faculty of Natural Sciences and Engineering, University of Ljubljana, 1000 Ljubljana, Slovenia; barbara.blaznik@ntf.uni-lj.si (B.B.); tanja.medved@ntf.uni-lj.si (T.M.); 2 University of Zagreb Faculty of Graphic Arts, 10000 Zagreb, Croatia; dorotea.kovacevic@grf.unizg.hr (D.K.); pbuljat23@unizd.hr (P.B.); iva.sarcevic@grf.unizg.hr (I.Š.); maja.brozovic@grf.unizg.hr (M.B.); 3 Department of Applied Communication Studies, University of Zadar, 23000 Zadar, Croatia

**Keywords:** appeal, eye-tracking, pictogram, typography, visual design

## Abstract

This study investigated how congruence between pictogram complexity and typographic complexity influences visual processing and perceived appropriateness in multimodal communication. Drawing on theories of visual communication, legibility and aesthetic perception, the study examined whether simple or complex pictograms harmonise more effectively with sans-serif or serif typefaces. Ninety participants viewed stimuli from three thematic categories (cobbler, herbal pharmacy and gluten-free restaurant), while their eye movements were recorded using a Tobii Pro Fusion eye-tracking device. Measures included reading time, fixation count and saccade count, together with subjective evaluations of pictogram–typeface suitability. The results show that reading time was the most sensitive indicator of formal congruence. In the cobbler and gluten-free restaurant categories, simple pictograms increased reading time with sans-serif typography but decreased it with serif typography. In the herbal pharmacy category, simple pictograms and sans-serif typography independently supported faster reading performance. Subjective evaluations showed no significant differences between combinations, indicating that participants perceived all pairings as similarly appropriate despite measurable differences in processing efficiency. The findings suggest that the effectiveness of pictogram–typeface combinations depends on both formal complexity and thematic context. Eye-tracking proved valuable for revealing subtle cognitive processing differences not reflected in subjective judgements.

## 1. Introduction

Pictograms and alphabetic writing represent two fundamental forms of visual communication [1,2,3].

In general, pictographic symbols can be “read” without knowledge of the creator’s spoken language. These simplified visual representations, or pictograms, communicate through stylised depictions of familiar objects in an analogical or figurative manner [2,3,4,5,6,7]. However, pictograms are not universally understood; their interpretation may vary significantly due to differences in culture, age, lifestyle, and subject familiarity. It has been shown that ethnic background influences the perception of graphic symbols across different groups, and that individuals from diverse cultural and linguistic environments do not interpret symbols uniformly [8]. Consequently, pictogram design requires thorough evaluation of all potential limitations that may reduce effectiveness, including the demographic characteristics of the target audience [9,10]. Graphic designers must therefore consider these variables to create pictograms that communicate effectively with intended users [11]. Specific design details are essential to ensure clear symbol recognition. Thoughtful pictogram design enriched with thematic information is crucial for effective communication [12]. Previous eye-tracking studies on pictograms have provided valuable insights into visual attention and processing [13,14,15]. However, many of these studies rely on a limited range of pictogram themes, making it difficult to determine whether the eye-tracking findings apply to pictograms representing more diverse themes and categories. To address this limitation, the present study examined pictograms from three distinct thematic categories, thereby covering a broader range of visual characteristics.

Visual appeal can significantly influence how people perceive and use visual content, often enhancing perceptual enjoyment and generating positive emotional responses [16]. A study on signage consistently calls for ongoing improvements in visual communication to make pictograms more intuitive and broadly accessible [17]. This is supported by studies emphasising the importance of pictograms in facilitating communication and comprehension across diverse cultural contexts [18,19,20]. Effective visual design consequently evokes positive emotional responses, highlighting the importance of good design [21]. Although precise measurement of visual attractiveness remains challenging, several studies have successfully explored related aspects, such as correlations between aesthetic qualities and perceived visual appeal [22,23,24].

Alphabetic writing systems are distinguished by their capacity to represent nearly all spoken content using a relatively small set of letters, each corresponding to specific sounds. However, understanding alphabetic writing requires knowledge of the writer’s language [1,3,25]. Typeface design has therefore become a central concern in legibility studies [25,26]. Letterform shape, overall typographic design, type size, and colour choice all affect text legibility and the clarity of information presentation [27,28,29,30,31,32]. Improved legibility encourages more positive attitudes towards text, thereby enhancing comprehension and memory retention [33,34,35,36,37]. Studies have examined whether simpler typefaces are more legible than complex ones. Findings suggest that simpler forms can reduce visual noise; however, excessive geometric simplification may lead to confusion between individual characters [26,38,39]. Letterforms must hence maintain clear, distinctive shapes with sufficient counter sizes [38,39,40]. Highly complex typefaces, e.g., decorative typefaces, generally reduce the legibility of longer texts [40].

Studies have also investigated whether simple or complex forms are more effective in pictograms and typography. Results indicate that simplicity alone does not necessarily improve comprehension [41]. While organised complexity may enhance usability, disorganised complexity can negatively affect immediate recognition and memory [42]. Appropriate typographic complexity may improve visual presentation [32]. Studies have further shown that simpler pictogram and typographic styles may be more suitable for older users [43]. In some cases, simplified typography may surpass pictograms in comprehensibility, while pictogram effectiveness depends strongly on clarity and contextual relevance [44,45]. Moreover, users appear to be subjectively sensitive to both the aesthetic and structural properties of visual representations, including variations in visual complexity [46,47]. Recent findings indicate that typeface selection can shape subjective evaluations of visual appearance, even when objective legibility remains unaffected [48]. Typography may therefore influence users’ overall impressions and preferences when evaluating visual material. In contrast, an earlier study suggests that shape characteristics may play a more critical role than typeface characteristics in subjective preference [49]. Taken together, these findings suggest that both pictorial and textual attributes contribute to the subjective evaluation of visual materials. Nevertheless, it remains unclear how these attributes are perceived in combination. Therefore, the present study examined how the visual coherence of pictogram–typeface combinations is subjectively perceived as a function of structural complexity. The aim of the study was to determine how combinations of simple or complex pictograms with simple or complex typography interact, and what influence these combinations have on subjective perception. The first hypothesis focuses on objective indicators of visual attention and assumes significant differences in eye-tracking measures between pictogram–typeface combinations with different levels of visual complexity. The second hypothesis concerns subjective perception and predicts significant differences in the subjective evaluation of pictogram–typeface combinations with different levels of visual complexity.

## 2. Materials and Methods

**Figure 1:**
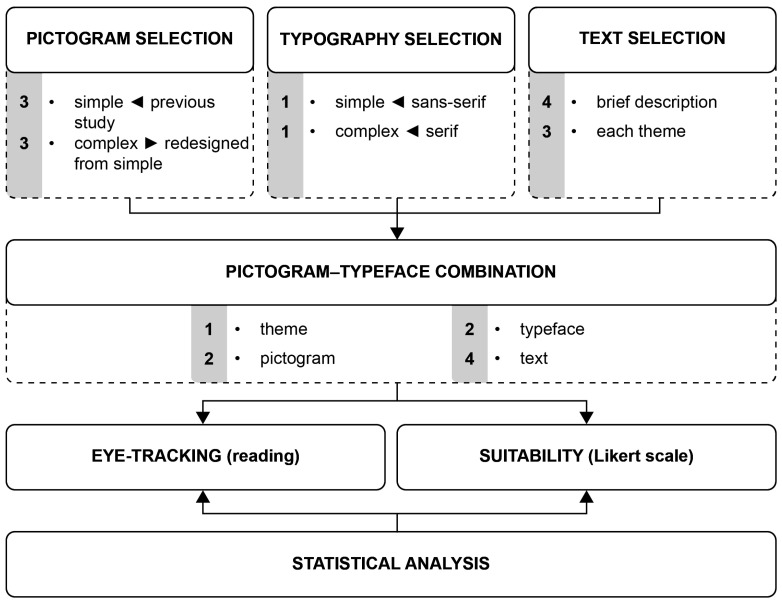
Experimental plan.

The experimental plan for the study is shown in Figure 1, which illustrates the sequential phases of the study. These comprise the design phase, covering the selection of pictograms, typography and text; the analysis phase, which measures reading speed for different types of pictograms and typefaces, and evaluates user preferences using a Likert scale; and the final phase, which consists of the statistical analysis and interpretation of the results.

### 2.1. Stimuli

Three thematic categories were used in this study, each representing a specific service category: a cobbler, a herbal pharmacy and a gluten-free restaurant. For each category, two versions of pictograms differing in visual complexity were prepared, i.e., a simple version and a complex version. Each version was paired with two typefaces, a sans-serif and a serif typeface, resulting in four stimulus variants per category. The simple pictograms used in this study were adopted from a previous study [50], in which their interpretation accuracy exceeded the minimum threshold set by the ISO 9186-1 standard [51] for acceptable pictogram comprehension. The complex versions were developed based on the corresponding simple versions. The core motifs were preserved, while additional graphic elements and visual details were introduced to increase the overall visual complexity. The simple versions consisted exclusively of solid black shapes. The modifications used to develop the complex versions were implemented as follows. In the cobbler pictogram, line-based elements were introduced alongside the solid forms, resulting in a combination of outlined (unfilled) and solid-filled elements. In the herbal pharmacy pictogram, the container was redesigned with additional detail in both the cap and the leaves, achieved through a combination of outlined and solid-filled elements. In the gluten-free restaurant pictogram, outlined and solid-filled elements were combined in the depiction of the knife and spoon, and fine lines were added to the wheat grain to enhance its immediate recognition.

To ensure visual consistency across all three pictograms, the same design principles were applied throughout. Uniformity was achieved by maintaining consistent line weight, a uniform black-and-white fill style, standardised bounding proportions and comparable figure–ground relationships. The introduction of subtle three-dimensional cues was also applied consistently across all motifs. As a result, the complex pictograms formed a coherent visual set and within each category, the complex version could be perceived as a refined variant of the simple one rather than as a distinct depiction. The visual complexity of each pictogram was characterised by the presence of the following structural features: outlined forms, negative space strokes and depth cues (Table 1). Outlined forms were defined as closed, unfilled shapes delimited by a visible contour. Negative space strokes were defined as white internal line details. Depth cues were defined as spatial or three-dimensional visual elements, suggesting depth.

The decision to enrich the pictograms with additional figurative detail was supported by prior studies, which indicated that more concrete and visually detailed representations tend to be more easily understood than more abstract ones. Collaud et al. [46] found that concreteness, defined as the extent to which an icon resembles a real-world object, has a strong and significant effect on icon comprehension, with more concrete icons being substantially more likely to be correctly interpreted. Similarly, Liu et al. [52] reported that concrete icons are recognised more quickly, more accurately and with a lower cognitive load compared to more abstract ones. These findings support the rationale that increasing visual detail in the complex pictograms could enhance their immediate recognition. Additional testing of complex pictograms was thus not considered necessary for the purposes of the present study.

Each pictogram was presented with a brief textual description of its depicted theme. All texts were written in a simple, comprehensible language. For each thematic category, four different text variants were prepared, each containing 90 to 110 characters. The texts contained 35 to 44 vowels, corresponding to a vowel proportion of 38–42% of the total number of letters [53]. To facilitate reading, the short texts were divided into lines according to meaning.

Based on the design characteristics of the pictograms, two typefaces were selected to complement them aesthetically. These typefaces were chosen for their differing levels of formal complexity, allowing the examination of how typographic style may influence viewers’ perception and interpretation of the stimuli. Typefaces with variation in stroke thickness, different forms of serifs and varying axes of stroke contrast are generally considered more complex. Conversely, typefaces with minimal or no variation in stroke thickness and without serifs are typically classified as simpler typefaces [26,28]. The first selected typeface was the geometric sans-serif Montserrat [3], which represents a visually simpler typographic structure, characterised by relatively uniform stroke widths, absence of serifs, angular stroke endings and a predominantly vertical axis. This modern typeface is distinguished by the humanistic form of the lowercase “a”, larger counters, larger x-height, and is considered to have satisfactory legibility for longer texts. In contrast, Chaparral Pro exhibits greater visual complexity through its pronounced serifs, varying stroke widths and inclined axis. It has pronounced, angular serifs characteristic of slab serif typefaces, while its letters are distinguished by soft transitions between strokes, differences in stroke weight and an inclined axis, which are features of old-style typefaces [3]. The combined use of both typographic styles ensured consistent legibility across different text sizes. The inclusion of these contrasting typographic styles was motivated by previous studies, which showed that letterform characteristics and typeface likeability can evoke different emotional responses in readers and influence reading experience, fluency, perception and cognitive processing during reading [36,37].

### 2.2. Equipment and Participants

Eye movements were recorded using a Tobii Pro Fusion eye-tracking device (sampling rate: 250 Hz; maximum gap duration: 75 ms; velocity threshold: 30°/s) in combination with Tobii Pro Lab 1.232 (x64) software (Tobii AB, Danderyd, Sweden). The device was cleaned with 70% isopropyl alcohol after each participant. The system tracks eye movements by detecting corneal reflections produced by infrared illuminators positioned at the front of the device. These illuminators generate characteristic light patterns reflected on the cornea, which are recorded by an infrared-sensitive camera to track fixations and eye movements [54]. For each stimulus, a single area of interest (AOI) was defined to include both the pictogram and the accompanying text. Visual stimuli were presented on an LCD monitor with a resolution of 2560 × 1440 px and a refresh rate of 60 Hz.

All measurements were conducted in a quiet room with neutral matte grey walls in compliance with ISO 3664 [55]. Participants were seated at a viewing distance of 60 cm ± 1 cm from the display in line with the recommendations of ISO 9241-303 [56].

**Figure 2:**
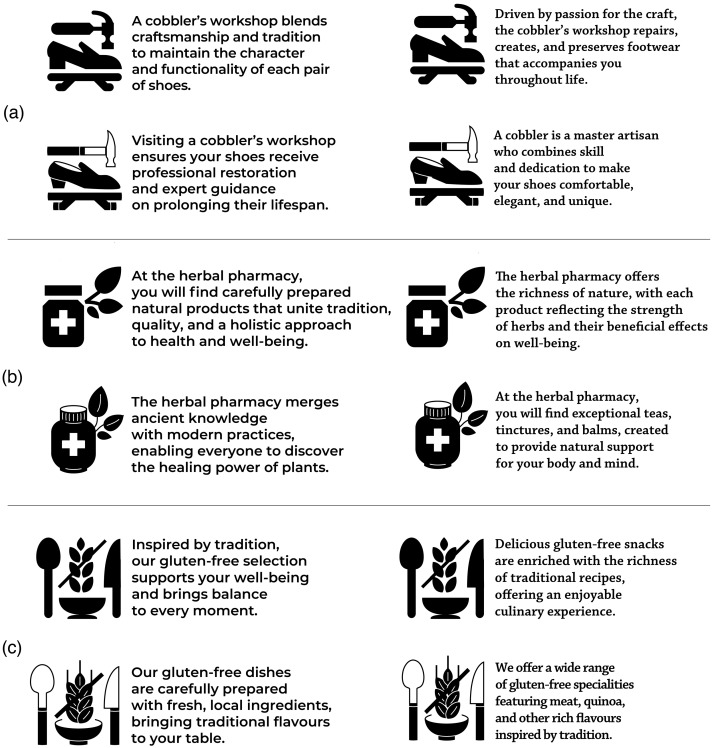
Simple (top) and complex (bottom) pictograms paired with sans serif (left) and serif (right) typefaces for (**a**) cobbler, (**b**) herbal pharmacy and (**c**) gluten-free restaurant, with accompanying texts translated into English.

In the tests, the text was arranged in five lines beside the pictogram. The original Slovenian text is provided in the Appendix A, and the English translation is shown in Figure 2. To standardise the visual appearance, slightly different type sizes were used for Montserrat (Medium) and Chaparral Pro (Semibold) (Table 2). The pictogram sizes are shown in Table 2. Both the typefaces and the pictograms were presented in black (#000000) on a white background (#ffffff).

The study included 90 participants aged 19 to 25 years (*M* = 21.33); 68.89% were female and 31.11% male. A relatively homogeneous sample of young adults was selected to reduce interindividual variability due to age-related differences in visual perception and reading performance. Participants were assigned to three groups of 30 individuals. All participants had normal or corrected-to-normal vision and were either native speakers of Slovenian or held official certification of proficiency in Slovenian. Table 3 presents the participants’ age data.

### 2.3. Procedure

Prior to the experimental session, each participant was informed of the purpose of the study, the procedures to be followed and their specific tasks. Before data collection, participants completed a five-minute adaptation period to the ambient lighting conditions. This was followed by a nine-point screen-based calibration procedure to ensure accurate gaze recording.

Each participant read four different texts in two typefaces. Each text was presented with two pictogram designs (simple and complex versions). To minimise fatigue effects, the reading order was counterbalanced using a Latin square design.

During reading, eye-tracking measurements were collected for each text across all participants, including reading speed, fixation count and saccade count. After each text, participants answered a comprehension question by indicating whether the statement was true or false.

After completing the reading task, participants were presented with all four designs, each representing a different combination of pictogram and typeface complexity. Then, they rated the suitability of the pictogram–typeface combination on a seven-point Likert scale.

To ensure comparability across texts, reading speed, fixation count and saccade count were normalised per 100 characters. As word length varies across languages, character count rather than word count was used as the primary unit of measurement, following Trauzettel-Klosinski et al. [57], to enhance the generalisability of the findings.

The effects of the typeface and pictogram design on legibility were analysed statistically using SPSS Statistics 17 (SPSS, Chicago, IL, USA). Repeated-measures analyses of variance (ANOVA) were conducted, with all statistical hypotheses tested at a significance level of 0.05. The Friedman test was used to assess the differences in perceived visual coherence of the pictogram–typeface combination.

## 3. Results

Results are presented for three participant groups, each exposed to one thematic category: a cobbler (group 1), a herbal pharmacy (group 2) and a gluten-free restaurant (group 3). For all analyses, only trials in which participants provided the correct answer in the text comprehensibility task were included (cobbler: *N* = 20; herbal pharmacy: *N* = 24; gluten-free restaurant: *N* = 25). This criterion was applied to limit the influence of responses that may have reflected insufficient attention to the reading task. To examine the effects of pictograms and typeface on the dependent variables, separate 2 × 2 repeated-measures ANOVAs were conducted for each theme. The two within-subject factors were pictogram complexity (simple versus complex) and typeface complexity (sans-serif versus serif).

Gaze plots for each participant were visually inspected to verify that the viewing strategy did not indicate dispersed attention or visual confusion that would suggest difficulties in understanding the pictograms.

**Figure 3:**
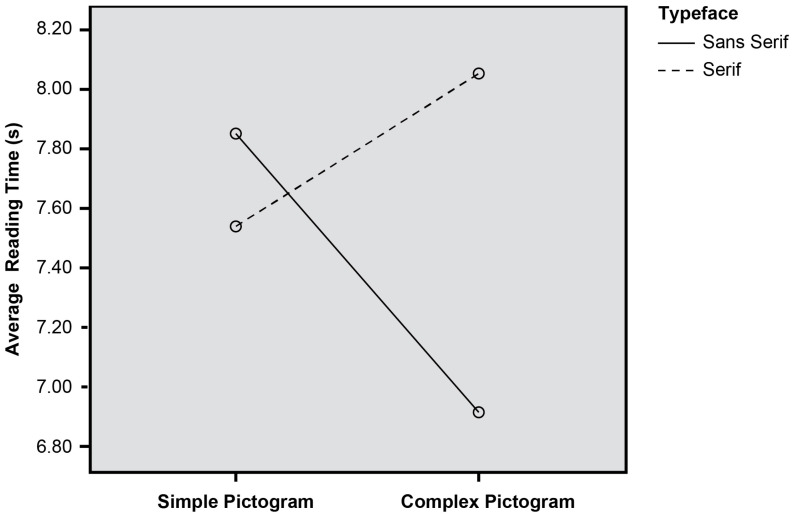
Interaction between pictogram complexity and typeface on reading time in cobbler thematic category.

**Figure 4:**
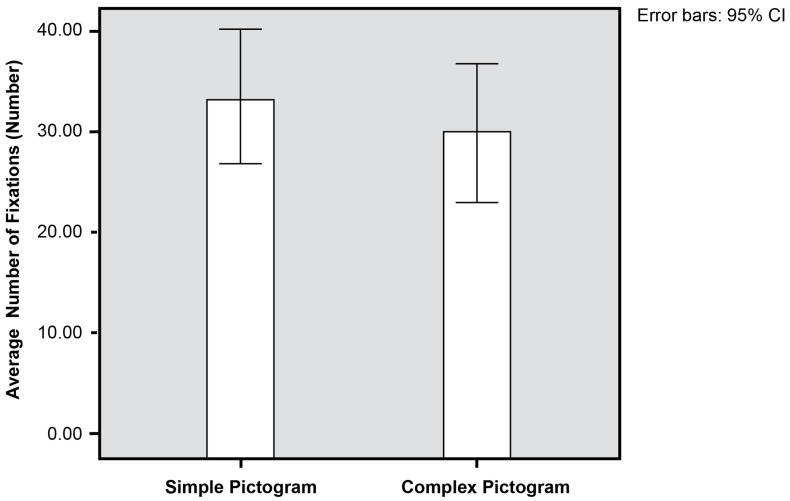
Number of fixations for stimuli with simple and complex pictograms in cobbler thematic category.

For reading time in the cobbler category, there was a significant interaction between pictogram complexity and typeface, *F*(1, 19) = 7.57, *p* < 0.05, *η*^2^*p* = 0.285, indicating that the effect of pictogram complexity on reading time depended on the typeface. The interaction contrast was 1.42 s (95% CI [0.34, 2.50]). Specifically, for the sans-serif typeface, reading time was higher for the stimuli with simple pictograms (*M* = 7.83, *SD* = 1.43) than for the stimuli with complex pictograms (*M* = 6.91, *SD* = 1.51), whereas for the serif typeface, reading time was lower for the stimuli with simple pictograms (*M* = 7.55, *SD* = 2.01) than for those with complex pictograms (*M* = 8.05, *SD* = 1.96). This interaction is shown in Figure 3. In the cobbler category, another significant result was found for the number of fixations. A significant main effect of pictogram complexity was observed, *F*(1, 19) = 4.97, *p* < 0.05, *η*^2^*p* = 0.207, with a higher number of fixations for the stimuli with a simple pictogram (*M* = 33.37, *SD* = 13.09) than for the stimuli with a complex pictogram (*M* = 29.99, *SD* = 13.49). The difference between the two levels is shown in Figure 4. In the cobbler category, all remaining main effects and interactions were not statistically significant (all *ps* > 0.05). Table 4 summarises the repeated-measures ANOVA results for the cobbler thematic category.

**Figure 5:**
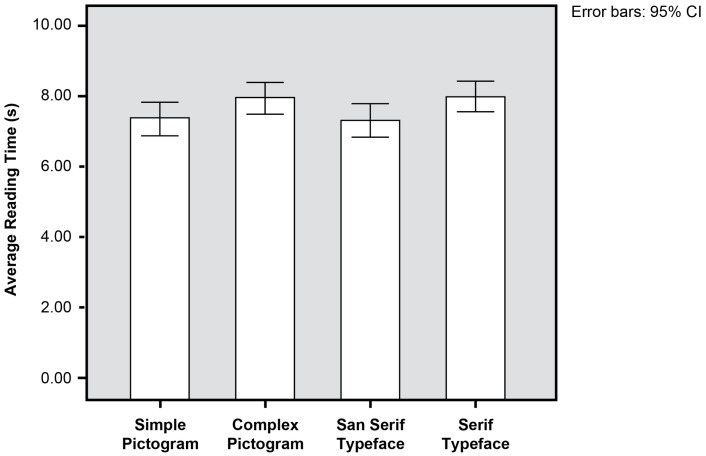
Average reading time across conditions in herbal pharmacy thematic category.

In the herbal pharmacy thematic category, a significant main effect of the typeface was found for reading time, *F*(1, 23) = 5.81, *p* < 0.05, *η*^2^*p* = 0.202, with higher values for the stimuli presented with the serif typeface (*M* = 7.95, *SD* = 0.95) than for the stimuli presented with the sans-serif typeface (*M* = 7.32, *SD* = 1.03), corresponding to a mean difference of 0.63 s (95% CI [0.09, 1.16]). Moreover, a significant main effect of pictogram complexity was found, *F*(1, 23) = 4.60, *p* < 0.05, *η*^2^*p* = 0.167, with lower values for the stimuli with a simple pictogram (*M* = 7.35, *SD* = 1.03) than for the stimuli with a complex pictogram (*M* = 7.91, *SD* = 0.95). The mean difference between complex and simple pictograms was 0.56 s (95% CI [0.02, 1.09]). Figure 5 illustrates the main effects of pictogram complexity and typeface on reading time. No other main effects or interactions reached statistical significance (all *ps* > 0.05) in the herbal pharmacy category. Table 5 summarises the repeated-measures ANOVA results for this category.

**Figure 6:**
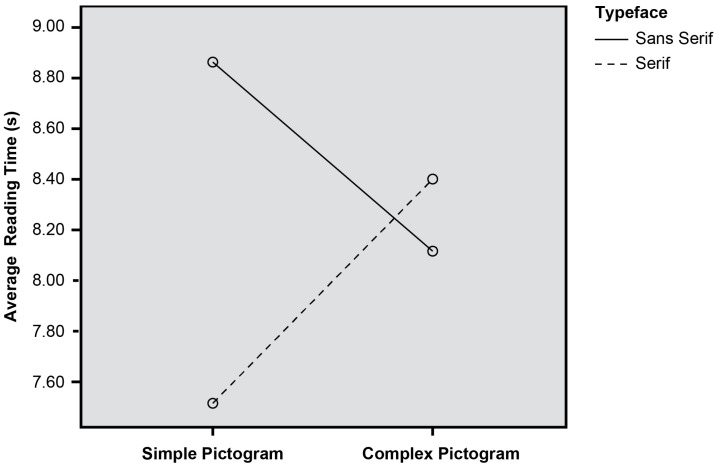
Interaction between pictogram complexity and typeface on reading time in gluten-free restaurant thematic category.

In the gluten-free restaurant category, only one statistically significant effect was observed across all analyses (Table 6). Specifically, an interaction between pictogram complexity and typeface was found for reading time, *F*(1, 24) = 12.19, *p* < 0.01, *η*^2^*p* = 0.337. In the sans-serif condition, reading time was higher for simple than for complex pictograms, with a mean difference of 0.75 s (95% CI [0.02, 1.48]), whereas in the serif condition, reading time was lower for simple than for complex pictograms, with a mean difference of −0.89 s (95% CI [−1.54, −0.24]). The interaction contrast was 1.64 s (95% CI [0.67, 2.62]). Figure 6 shows this interaction.

The differences in the perceived visual coherence of the pictogram–typeface combination were analysed using the Friedman test. As shown in Table 7, the results indicated that the differences between conditions were not statistically significant (all *ps* > 0.05). These findings suggest that across all three thematic categories (a cobbler, a herbal pharmacy and a gluten-free restaurant), the pictogram–typeface combinations were perceived as equally appropriate, regardless of pictogram complexity or typeface.

## 4. Discussion

The aim of this study was to determine which combinations of pictogram and typeface complexity facilitate faster information processing when information is conveyed through a combination of text and image. Specifically, across three stimulus categories (cobbler, herbal pharmacy and gluten-free restaurant), we examined the influence of pictogram and typeface on reading time, fixation count and saccade count, while also considering participants’ perceived visual coherence of each pictogram–typeface combination.

Participants’ subjective judgements of pictogram–typeface combinations showed no statistically significant effects, indicating that the second hypothesis was not supported. This suggests that participants perceived all combinations as similarly appropriate and that none of the tested pairings were judged more favourably than the others. This finding is consistent with previous studies indicating that relatively subtle formal variations in the combination of visual and verbal information do not necessarily lead to differences in subjective responses. For example, Vecino et al. [58] reported no significant differences in user preferences when comparing typefaces with different structural characteristics, specifically serif and sans-serif variants within the same typeface family. Similarly, Zhu et al. [59] found that concrete visual elements paired with concrete textual elements may be processed as functionally equivalent, suggesting that some combinations of visual and textual features are perceived similarly. Nevertheless, subjective ratings, unlike eye-tracking data, may lack the sensitivity required to detect subtle perceptual or processing differences, as they rely on retrospective evaluations [60] rather than on moment-to-moment visual processing. Participants may therefore not have been consciously aware of small variations in visual compatibility or legibility that nonetheless influenced early perceptual processing.

Reading time was the only dependent variable that showed a statistically significant effect across all three themes. In the cobbler and restaurant thematic categories, a significant interaction between pictogram complexity and typeface was found for reading time. Across both categories, the effect of typeface depended on pictogram complexity, suggesting that the influence of the typographic style was not uniform but varied with the visual context in which the text was presented. In the present study, reading time with the sans-serif typeface was longer for the designs containing simple pictograms than for those containing complex pictograms. With the serif typeface, however, reading time was shorter for the designs with simple pictograms than for those with complex pictograms. The interaction between pictogram complexity and typeface may reflect the importance of semantic or stylistic coherence. Rather than responding independently to each visual component, participants may have processed the pictogram and accompanying text as an integrated visual message. Congruent combinations may facilitate perceptual fluency, whereas incongruent combinations may require additional processing despite similar subjective evaluations [60]. These findings extend previous studies on the design of pictograms and typography by demonstrating that the effect of pictogram complexity is contingent upon typographic style, indicating an interaction between pictorial and textual visual complexity [45,46].

The findings further suggest that context dependence may arise only under certain thematic conditions. Unlike the interaction observed in the cobbler and restaurant categories, no interaction was found in the pharmacy category. This indicates that, in this category, the role of typeface was relatively stable across the two levels of pictogram complexity. A possible explanation is that the interaction between the typeface and pictogram complexity may depend on additional properties of the thematic context.

At the same time, the results observed in the pharmacy thematic category indicated separate effects of typeface and pictogram complexity on reading time. The designs presented in a serif typeface were read more slowly than those in a sans-serif typeface, while the designs containing a simple pictogram were read more quickly than those with a complex pictogram. Complex pictograms contain a greater number of visual features, which may compete for attentional resources. According to theories of visual salience, visually prominent objects attract early fixations irrespective of task relevance. When the accompanying text also requires processing, highly detailed pictograms may compete with textual information, increasing overall reading time [61]. The slower reading times observed for serif typefaces may reflect greater visual complexity associated with more detailed letterforms. This interpretation is consistent with studies showing that visually complex typographic styles can increase perceptual processing demands and reduce reading fluency [28,32,40]. Although previous findings regarding serif and sans-serif legibility remain mixed [29,38], several studies have emphasised that typographic detail and visual complexity can influence reading efficiency, particularly in digital reading environments [36,37].

One of the more distinctive findings of this study was the significant effect observed for the number of fixations within the cobbler category. In this category, stimuli containing simple pictograms elicited more fixations than those containing complex pictograms, suggesting that reducing pictogram complexity did not necessarily lower visual processing demands. One possible explanation is that the cobbler category represented a less familiar semantic domain for the participants than the pharmacy and restaurant categories [62]. However, in a previous study involving participants from different age groups [50], the cobbler pictogram demonstrated a high level of recognisability. This discrepancy suggests that familiarity with the semantic category, rather than with the pictogram itself, may have influenced visual processing in the present study. Consequently, participants may have required additional fixations to verify the meaning of even visually simple pictograms. This interpretation is consistent with a previous study on user interfaces, which has shown that visual elements that are not immediately interpretable increase the effort required for visual processing [63].

Taken together, these findings indicate that the first hypothesis was partially supported and highlight the importance of considering both pictogram complexity and typeface when designing text–picture combinations. By showing that these effects can differ across thematic categories and dependent measures, the study suggests that no single combination of pictogram and typeface is likely to be optimal in all visual design settings.

This study has several limitations that should be considered when interpreting the findings. Firstly, the participant sample consisted exclusively of young adults aged 19 to 25 years. As visual processing, aesthetic preferences and familiarity with pictographic and typographic styles may vary across age groups, educational backgrounds and cultural contexts, the generalisability of the results is limited. Future research should therefore include more diverse participant populations to examine whether the observed effects, particularly interaction patterns between pictogram and typeface complexity, extend to broader demographic groups. Secondly, only three thematic categories were investigated, which may not fully capture the variability in real-world visual communication contexts. Since the present findings suggest that contextual factors influence the relationship between pictograms and typography, additional thematic domains should be included in future studies to test the robustness of these context-dependent effects. Thirdly, the selection of typefaces was not preceded by systematic pre-testing to establish perceived levels of typographic simplicity or complexity. Future research should thus validate a broader set of typefaces using perceptual ratings to ensure a more robust manipulation of typographic complexity. In addition, the textual material used in the experiment was relatively short. Although appropriate for controlled experimental conditions, longer text passages (e.g., more than 200 characters) may provide more stable measures of reading behaviour and improve the sensitivity of eye-tracking indicators. A further methodological limitation concerns the exclusion criterion used to ensure task compliance, which reduced the final sample size and may have affected statistical power, particularly for detecting interaction effects. Future studies should hence plan for a larger initial sample to compensate for potential data loss. Finally, subjective coherence ratings may not have captured subtler aesthetic or affective responses to the stimuli. Future research should consider incorporating additional subjective measures, as well as more diverse visual and textual stimuli to better capture the complexity of multimodal perception.

## 5. Conclusions

This study demonstrates that the visual processing of pictogram–typeface combinations depends not only on the individual characteristics of pictograms and typefaces, but also on the interaction between these elements and the semantic context in which they are presented. Reading time emerged as the most sensitive measure of processing efficiency, revealing that the effects of pictogram complexity were contingent on typographic style in some thematic categories, whereas in others, pictogram and typeface complexity exerted independent influences. These findings indicate that the relationship between pictorial and typographic design is context-dependent rather than governed by a single universal principle.

The absence of differences in participants’ subjective evaluations, despite measurable differences in eye-movement behaviour, further suggests that users are not always consciously aware of design characteristics that influence cognitive processing. Evaluations based solely on subjective preferences may therefore fail to capture important aspects of communication efficiency.

Taken together, the findings support the view that pictograms and accompanying text are processed as an integrated visual message rather than independent design elements. The effectiveness of multimodal visual communication therefore depends on the interplay between pictorial complexity, typographic characteristics and semantic context. This perspective extends current understanding of visual communication by demonstrating that formal design variables influence information processing through their interaction rather than solely through their individual effects.

From a practical perspective, the findings suggest that the designers of public information systems, signage, branding and other forms of integrated visual communication should consider pictograms and typography as components of a unified communication system. Rather than applying general design rules, the selection of pictogram and typeface combinations should take into account the semantic context in which information is presented and the characteristics of the intended audience.

Future research should examine how semantic familiarity, cultural background and user characteristics interact with pictogram and typographic complexity to shape visual processing. Investigating a broader range of communication contexts and participant populations would help establish the extent to which the context-dependent relationships observed in this study can be generalised across different visual communication environments.

## Figures and Tables

**Table 1:** Presence of structural complexity features for simple and complex pictograms (0 = absent; 1 = present).

Theme	Pictogram Complexity	Outlined Forms	Negative Space Strokes	Depth Cues
Cobbler	Simple	0	0	0
Cobbler	Complex	1	1	1
Herbal pharmacy	Simple	0	0	0
Herbal pharmacy	Complex	1	1	1
Gluten-free restaurant	Simple	0	0	0
Gluten-free restaurant	Complex	1	1	1

**Table 2:** Technical specifications of test stimuli.

Pictogram Size by Theme	Simple	Complex
Cobbler	246 × 231 px	246 × 228 px
Herbal pharmacy	246 × 239 px	260 × 217 px
Gluten-free restaurant	244 × 235 px	252 × 244 px
**Typeface size**	40 pt	41 pt

**Table 3:** Age data of participants in each testing group.

Number of Participants Age (19–25 Years)	90	*M* 21.33	*SD*
Group 1	30	21.23	1.09
Group 2	30	21.67	1.06
Group 3	30	21.10	0.98

**Table 4:** Summary of repeated-measures ANOVA results for cobbler thematic category.

Measure	Effect	*df* (Effect, Error)	*F*	*p*-Value	*η* ^2^ *p*
Reading time	Typeface	1, 19	1.49	0.24	0.073
Reading time	Pictogram	1, 19	1.23	0.28	0.061
Reading time	Typeface × Pictogram	1, 19	7.57	0.01	0.285
Fixation count	Typeface	1, 19	0.03	0.86	0.002
Fixation count	Pictogram	1, 19	4.97	0.04	0.207
Fixation count	Typeface × Pictogram	1, 19	0.04	0.84	0.002
Saccade count	Typeface	1, 19	0.01	0.94	0.001
Saccade count	Pictogram	1, 19	1.39	0.25	0.068
Saccade count	Typeface × Pictogram	1, 19	0.14	0.71	0.007

**Table 5:** Summary of repeated-measures ANOVA results for herbal pharmacy thematic category.

Measure	Effect	*df* (Effect, Error)	*F*	*p*-Value	*η* ^2^ *p*
Reading time	Typeface	1, 23	5.81	0.02	0.202
Reading time	Pictogram	1, 23	4.60	0.04	0.167
Reading time	Typeface × Pictogram	1, 23	0.00	0.97	0.000
Fixation count	Typeface	1, 23	0.27	0.61	0.012
Fixation count	Pictogram	1, 23	0.71	0.41	0.030
Fixation count	Typeface × Pictogram	1, 23	3.74	0.06	0.140
Saccade count	Typeface	1, 23	0.43	0.52	0.018
Saccade count	Pictogram	1, 23	0.22	0.64	0.009
Saccade count	Typeface × Pictogram	1, 23	3.38	0.08	0.128

**Table 6:** Summary of repeated-measures ANOVA results for gluten-free restaurant thematic category.

Measure	Effect	*df* (Effect, Error)	*F*	*p*-Value	*η* ^2^ *p*
Reading time	Typeface	1, 24	3.69	0.07	0.133
Reading time	Pictogram	1, 24	0.09	0.77	0.004
Reading time	Typeface × Pictogram	1, 24	12.19	0.00	0.337
Fixation count	Typeface	1, 24	0.00	0.98	0.000
Fixation count	Pictogram	1, 24	2.97	0.09	0.110
Fixation count	Typeface × Pictogram	1, 24	1.61	0.22	0.063
Saccade count	Typeface	1, 24	0.00	0.96	0.000
Saccade count	Pictogram	1, 24	1.75	0.19	0.068
Saccade count	Typeface × Pictogram	1, 24	3.29	0.08	0.121

**Table 7:** Friedman test results for perceived visual coherence of pictogram–typeface combination across categories.

Thematic Category	*N*	*χ* ^2^	*df*	*p*	Kendall’s *W*
Cobbler	20	5.25	3	0.15	0.088
Herbal Pharmacy	24	5.41	3	0.14	0.075
Gluten-free Restaurant	25	2.24	3	0.52	0.030

**Figure A1:**
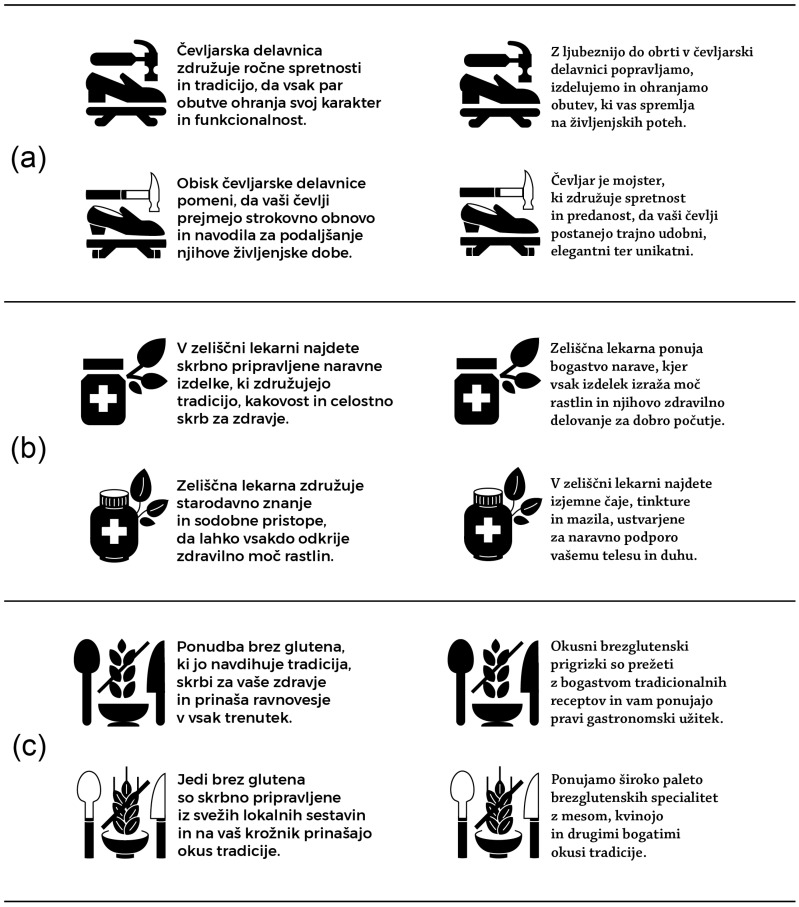
Simple (top) and complex (bottom) pictograms paired with sans-serif (left) and serif (right) typefaces for (**a**) cobbler, (**b**) herbal pharmacy, and (**c**) gluten-free restaurant.

## Data Availability

The data presented in this study are available upon reasonable request from the corresponding author. The data are not publicly available due to participants’ privacy.
